# Disaster management ontology- an ontological approach to disaster management automation

**DOI:** 10.1038/s41598-023-34874-6

**Published:** 2023-05-19

**Authors:** Deepika Shukla, Hiteshwar Kumar Azad, Kumar Abhishek, S. Shitharth

**Affiliations:** 1 Computer Science and Engineering, National Institute of Technology Patna, Patna, 800005 India; 2 School of Computer Science and Engineering, Vellore Institute of Technology Vellore, Vellore, 632014 India; 3 Department of Computer Science, Kebri Dehar University, 250 Kebri Dehar, Ethiopia

**Keywords:** Natural hazards, Environmental impact, Ecosystem services

## Abstract

The geographical location of any region, as well as large-scale environmental changes caused by a variety of factors, invite a wide range of disasters. Floods, droughts, earthquakes, cyclones, landslides, tornadoes, and cloudbursts are all common natural disasters that destroy property and kill people. On average, 0.1% of the total deaths globally in the past decade have been due to natural disasters. The National Disaster Management Authority (NDMA), a branch of the Ministry of Home Affairs, plays an important role in disaster management in India by taking responsibility for risk mitigation, response, and recovery from all natural and man-made disasters. This article presents an ontology-based disaster management framework based on the NDMA’s responsibility matrix. This ontological base framework is named as Disaster Management Ontology (DMO). It aids in task distribution among necessary authorities at various stages of a disaster, as well as a knowledge-driven decision support system for financial assistance to victims. In the proposed DMO, ontology has been used to integrate knowledge as well as a working platform for reasoners, and the Decision Support System (DSS) ruleset is written in Semantic Web Rule Language (SWRL), which is based on the First Order Logic (FOL) concept. In addition, OntoGraph, a class view of taxonomy, is used to make taxonomy more interactive for users.

## Introduction

A disaster can be caused by natural or man-made reasons, resulting in environmental degradation, the destruction of public and private property, and the loss of precious lives. Natural disasters killed approximately 45,000 people worldwide each year on average over the last decade^1^. This accounts for about 0.1% of all deaths worldwide. According to the considered statistics^2^, 68% of India’s land is prone to drought, 12% to floods, 8% to cyclones, and 60% to earthquakes, making India one of the most disaster-prone countries in the world, affecting overall 85% of Indian land and more than 50 million people. The National Disaster Management Authority^3^ (NDMA) came in the role of reducing or avoiding these terrific activities. This facilitates the framework that coordinates all the central ministries and government departments and distributes responsibilities at state and central government levels in the pre-disaster and post-disaster spheres. The pre-stage disaster management includes the preparedness and mitigation phase, whereas response and recovery fall into the post-disaster stage. Different disaster management plans and activities execute under these phases. The management plan is decided by considering hazard-specific guidelines for different disaster families. Responsibility distribution is a big challenge because the whole system depends on that matrix. Work efficiency can be maximized if a single platform tells the respective authority about their responsibilities. On the other hand, it is a backbone-breaking task to help disaster suffered people under government norms.

The current era of smart city infrastructure requires an intelligent emergency response system for pre- and post-disaster management activity. The present need is a robust disaster management system to help the cities’ communities have better pre and post-disaster phases. Smart cities are characterized by data generated by highly heterogeneous technologies in format, structure, and delivery mechanisms. There is a need for a unified model to handle the heterogeneous data for making decisions. Ontology^4^ can be used to create a unified model in an extensible and reusable manner. The paper presents an ontological approach as the semantic structure’s backbone, such as semantic integration and definition of disaster-related information. It will be described as a vocabulary by experts and inferred from the data by semantic analysis. The disaster information is presented as an ontological model to explain the disaster facts and is linked with each other to aid decision-making.

The motivation of this article is to design knowledge-enabled management to improve assistance and response management for different stages of disasters. The proposed work adopts an ontology-based approach to designing the Decision Support System (DSS) and gives a framework for directing different disaster stages in India. This framework provides leadership for making any stage more accessible and enables the decision-making process to be more manageable and machine-interpretable. Many research groups are exploiting Basic Formal Ontology^5^ (BFO) to define unified vocabulary and information integration in information science. Upper-level ontology is an ontology of very general terms, such as objects and properties, and it defines common relations across the entire domain. There are more than 250 ontology-driven endeavors around the world^6^. An ontological approach is presented here to design a knowledge base on disaster-related facts. The proposed knowledge base has been developed with the help of semantic web technologies to enable the knowledge within the Semantic Web^7,8^. It presents a semantic characterization and dynamic classification of disaster-related events and external information^9^.

The remainder of the paper is structured as follows: Section “Related work” discussed related work to present a study of existing disaster management ontologies. Section “Proposed framework” proposes a framework for presenting the methodology for the proposed ontology development. Section “Implementation and result analysis” describes the characteristics of the proposed ontology DMO for describing disaster-related facts. Section 5 discusses a complete implementation of DMO to present a different type of calamity by inferencing new knowledge from ontology. Finally, section 6 concludes the proposed work.

## Related work

This section represents an overview of the existing disaster ontologies related to the crisis management and hazard domains. Table 1 summarizes existing disaster ontologies with their different features, such as domain coverage, URI, formalization, documentation, classes and relations, and evaluation. Wei Xu and Sisi Zlatanova^10^ proposed an approach to develop ontologies for disaster management response, ensure interoperability of emergency services, and present appropriate information at the right time and place to be used for emergency response before that geographical information integration was restricted to keyword-based matching. Another article^11^ proposed a case study to develop a disaster management decision support system with two essential elements: situation-based simulation and a different multi-criteria model. Situation-based simulation models are associated with floods and nuclear disasters, whereas a multi-criteria model allows the user to prepare an emergency preparedness plan and identify potentially affected sites and relocation sites.

Grigori^12^ and his colleagues face difficulties in locating appropriate sensor information in real-time fusion, particularly in disaster management, where the flow of information is overwhelming, and sensor data must be easily accessible to non-experts (fire brigade officers). An Ontology-based knowledge integration framework for managing floods in Malaysia has been proposed to improve interoperability between agencies during the critical time of disaster. This study^13^ proposed a Knowledge Integration Framework (KIF) for managing floods in Malaysia with ontology as the backbone. A knowledge treasure for military decision support was designed and evaluated by Mishra and Jain^14,15^ and developed a military resource ontology and procedures as a learning model for better interoperability of the Indian military resources. Tiwari and colleagues^16,17^ designed a semantic model for IoT-based health care that includes SWRL rules for interacting with patients and health workers. The developed ontology was tested using various tools and published online. D. N. Ford and C. M. Wolf^18^ presented a digital twin-based community disaster management model for smart cities. The authors discussed the risks associated with the proposed model and future improvements.

Tan et al.^19^ have presented a study on how AI-enabled cities can protect people from natural hazards and disasters. The authors have also discussed the impact of adopting AI in cities and societies. Zaheer Allam and David S. Jones^20^ present the need for a standardized protocol for data sharing among stakeholders of smart cities for better cooperation and management in case of an event outbreak or disaster. Daekyo Jung and colleagues^21^ designed an intelligent disaster management system for wildfires as well as cold and heat waves. The author has collected data from an open API and employed AI algorithms to make a decision. Another article in which Alexandros Nikitas and colleagues^22^ present an AI, transportation, and smart city collaboration for urban mobility. The authors primarily focused on Connected and Autonomous Vehicles (CAVs), autonomous Personal and Unmanned Aerial Vehicles (PAVs and UAVs), and Mobility-as-a-Service (MaaS).

The literature review in this article provides insight into the application of ontology in disaster management, situation awareness, and emergency response. The use of ontology aids decision-making when dealing with heterogeneous data. The recent growth of smart city infrastructure requires intelligent post- and pre-disaster emergency response management. The proposed ontology uses National Disaster Management Plan (NDMP)^3^ (National Disaster Management Plan) developed by the National Disaster Management Authority, Government of India. It provides knowledge-enabled management and control to improve assistance and response management.

In Table 1, 21 Natural Disaster related ontologies are presented, with 7 features (Ontology Name, Domain Coverage, URI, Formal, Documentation, Classes & Relations, and Evaluation) analysed for each ontology. Some related studies were also discussed in order to investigate disaster management ontologies.

**Table 1:** A comprehensive analysis of existing natural disaster ontologies.

Ontologies	Domain coverage	URI	Formal	Documentation	Classes & relation	Evaluation	References
Empathi	Emergency management during hazard crisis	Yes	OWL	Yes	423 classes & 338 relations	Yes	Gaur et al.^23^ (2019)
Ontology task allocation & Management	Urban search & rescue	NA	OWL	No	NA	Yes	Saad et al.^24^ (2018)
OntoEmerge	Early warning system for emergency situation	NA	OntoUML	No	NA	Yes	Moreira et al.^25^ (2017)
Situation Awareness Ontology (SAO)	Situation awareness	NA	RDF	No	NA	NA	Pai et al.^26^ (2017)
TTIPP Methodology	Incident command system	NA	PNML	No	NA	NA	Fang et al.^27^ (2019)
Crisis Response Ontology (CROnto)	Crisis response situations	NA	OWL	No	NA	Yes	Bannour et al.^28^ (2019)
Geographical Entity Ontology (GeoMD)	Geographical disaster	Yes	OWL	No	241 classes & 151 properties	Yes	Bouyerbou et al.^29^ (2019)
Ontology for Flood	Flood Management	NA	OWL	No	NA	NA	Rodzi et al.^13^ (2016)
University Activity Ontology (UAO)	Location-based services	NA	RDF	NA	NA	NA	Bouyerbou et al.^29^ (2017)
OntoCity	Disaster Monitoring	Yes	OWL	No	130 classes & 62 properties	Yes	Alirezaie et al.^30^ (2017)
HARE Ontology	Humanitarian aid in emergency situation	NA	OWL	No	446 classes & 178 properties	Yes	Apisakmontri et al.^31^ (2016)
LandSlip Ontology	Verification and prediction of landslide hazards	NA	OWL	No	98 classes & 26 properties	Yes	Phengsuwan et al.^32^ (2020)
Situation Awareness Ontology	Disaster Management	NA	OWL	No	NA	NA	Smets et al.^33^ (2017)
Climate Crisis management	Crisis Management	NA	OWL	No	38 classes and 59 properties	OOPS	Kontopoulos et al.^34^ (2018)
Disaster Ontology	Risk and crisis-related events during disaster	NA	OWL	No	NA	Yes	Narayann Samy et al.^35^ (2019)
EQ_Predicton Ontology	Earthquake prediction ontology	Yes	OWL	No	9 classes & 4 properties	NA	Ramamonjisoa et al.^36^ (2012)
Situation Theory Ontology	Ontology for situation behaviour	Yes	OWL	No	37 classes & 32 properties	Yes	Kokar et al.^37^ (2009)
Event Ontology	Disaster response & relief coordination	NA	OWL	Yes	116 classes & 6 properties	Yes	Kar et al.^38^ (2018)
Hydrological sensor web Ontology	Flood management	NA	OWL	No	34 classes & 103 properties	Yes	Wang et al.^39^ (2018)
Earthquake Emergency (EEM) Ontology	Earthquake emergency evaluation & response	http://local/ontologies/eemYes	OWL	No	356 classes & 41 properties	Yes	Spalazzi et al.^40^ (2014)

## Proposed framework

**Figure 1:**
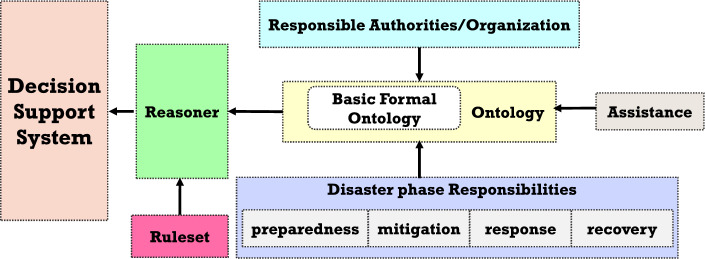
Proposed framework of DMO.

The Disaster Management Ontology (DMO) identified the need to remove ambiguity in the responsibility framework. Therefore, it particularizes who is responsible for what at different stages of understanding and handling the disasters. The DMO is always envisaged as being ready for activation in retaliation to an emergency. The proposed ontology is designed based on National Disaster Management Plan (NDMP). Figure 1 depicts the proposed framework for the development of DMO.

The creation of a formalized framework for the DMO program is categorized into two phases, namely:

- Phase 1: Static knowledge base using OWL
- Phase 2: Dynamic query environment using SWRL

The Knowledge Base (KB) is concerned with presenting information in a machine and user-friendly format. The semantic net, frames, rules, and ontologies are all used to represent knowledge. In this context, a semantic net (or semantic network) is a graph used to extract knowledge from natural language. The nodes in the graph represent the concepts, and the arc represents the relationship between those concepts. To express knowledge in simple and complex conditions, rules use the IF-THEN-ELSE statement. SWRL is used to infer knowledge created in the form of the DMO ontology. Essentially, rules are established based on facts. These facts are the condition of assistance provided by the state government to the suffered people. In disaster management, ontology is used to study what exists in the domain of interest and represent knowledge about this domain in a machine-readable format. The three main components of DMO are the concept, the relationship, and the individuals.

### Disaster management ontology (DMO)

DMO has been designed based on concepts acquired across disasters. Taxonomy, relations, and restrictions are framed among the entities to integrate with the decision support system. Disaster Management Ontology is a knowledge base that includes relevant concepts. Intrinsically, disaster responsibilities are divided into two categories: pre-disaster responsibility and post-disaster responsibility. Based on the behaviour and characteristics of related terms in these categories, BFO provides the platform for generalizing concepts. BFO believes that anything that exists must belong to some category of the entity group. The entity can be anything, including emotions, facts, objects, processes, qualities, models, etc. In BFO, continuant specializes all those entities that preserve their identity throughout the state of living. These entities preserve their identity even while obtaining or dropping the material part. In the DMO, the object is a class consisting of DMO-relevant entities. Satellite input plays a significant role in understanding risk. For this satellite, inputs are taken and disseminated to relevant agencies. The lead agency for this work is NDMA, SoI (Survey of India), NRSC (National Remote Sensing Centre), MoHA (Ministry of Home Affairs), and DoT^3^ (Department of Telecommunications). A satellite is a subclass of the object, and an object is a material entity that is extended in three dimensions and is maximally connected. For the realization of these entities, no further dependency is required.

**Figure 2:**
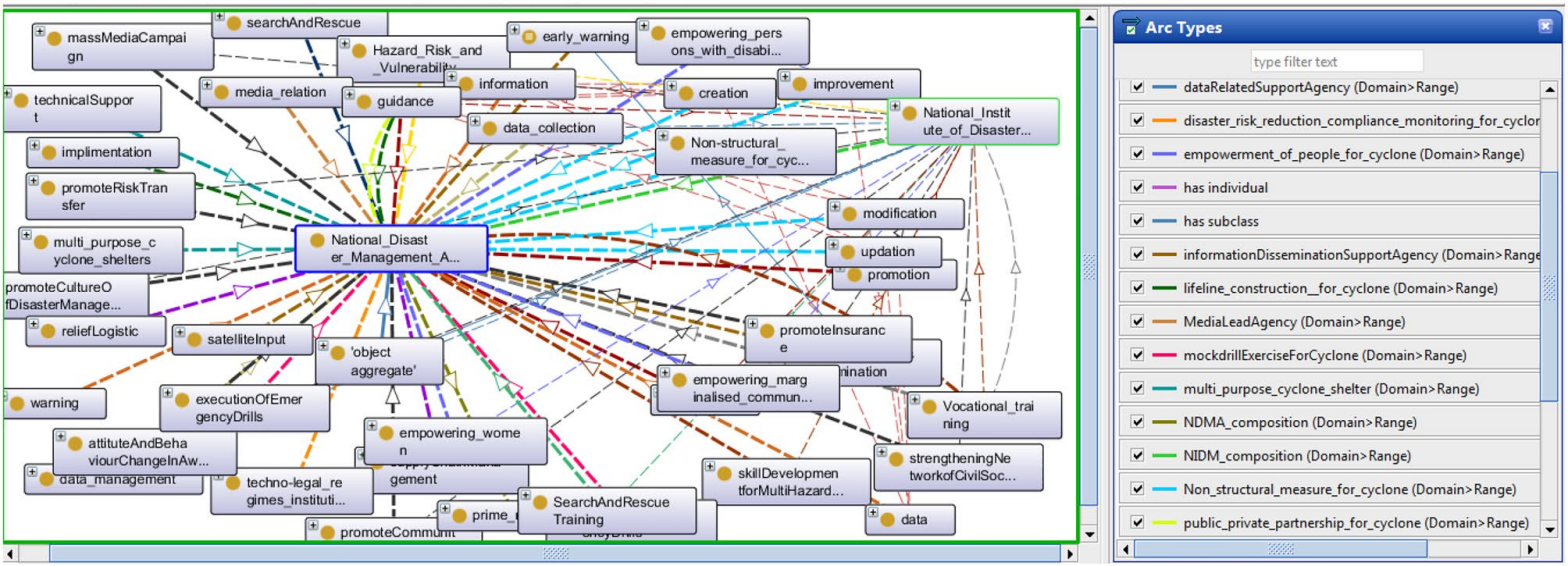
A snapshot from Protégé depicts NDMA dependency structure.

The DMO upper class of object aggregate includes all those entities whose body is made by many objects. These objects cannot share their parts in common but prolong with a distinct identity. By detaching any of these objects, the identity of the object aggregate cannot change. Here all the ministries of the Indian government, an organization like ISRO, forces such as the National Disaster Response Force and Air India Force, the community, Council, the NCC (National Cadet Crop), and central authority are filtered as object aggregate. The Ministry has a major responsibility for controlling any government action. Top-level decision-making authority^3^ for disaster management includes CCS (Cabinet Committee on Security), NCMC (National Crisis Management Committee), CAPF (Central Armed Police Force), and NIDM (National Institute of Disaster Management), NDRF (National Disaster Response Force), NDMA^3^. All these entities fall in this category. Figure 2 depicts the NDMA dependency graph, which shows how different activities related to disaster phases are dependent on their execution.

The North Indian Ocean is part of the ocean, and it is physically impossible to separate it from the rest of the ocean. Oceans are larger than the sea, but no one can separate one from the other. Similarly, a shallow water area cannot be separated from the remaining water area. The entities discussed above have been classified as fiat object parts. The entity under this class is a proper part of some larger object. It cannot be demarcated from the rest of the object by any physical discontinuities. A tsunami is connected with shallow water by showdown property, which denotes that the tsunami gets slowed down at shallow water level. Cyclone is connected through ‘causedBy’ property with the sea.

All entities bounded by any material entity in three-dimensional regions are classified as DMO upper-class sites. Houses, stations, centres, words, and states are in this class. The house is located in space and is bounded by a material entity. So, the existence of a house is fully dependent on its wall, so these entities are specifically dependent continuant. Specifically, dependent continuant is the category of BFO, completely dependent on its surrounding boundary.

A shoreline is the ornamental border of land at the edge of an enormous water body, such as an ocean, sea, or lake. The seashore can change position, shape, and magnitude as its material hosts move or change shape or size. It has been located in some spatial regions during its existence. It is of one dimension and does not include a spatial region. These entities are classified as a DMO upper class of Continuant Fiat Boundary.

Quality is something that all entities of a particular category have for all of the time they exist and does not demand any further activity to be perceived. In DMO, the quality class includes high frequency, highest flood level, etc. The term high frequency has been used for many purposes, such as VHF sets and satellite phones with very high frequency. Marking the highest flood level is a process in which areas with an elevated flood level are considered. The entities coming under the DMO upper class of the realizable entity category are exhibited only through certain characteristic realization processes. Their order of execution is realized. These entities have at least one independent continuant as their bearer.

In this ontology, the entities under the realizable category are role, policy, regulation, constitution, promotion, warning, etc. All these entities can only be realized during execution and depend on a single or a group of matter. It is further classified into many categories, mainly on the role and disposition. Many entities do not follow any of the mentioned classes; instead, they stay on the realizable class itself.

Disposition is an internally grounded realizable entity. Internal structure, environment, and factors are responsible for the existence and execution of these entities. The disposition is not a prerequisite but can occur at any instance. The proposed ontology contains diseases caused by different factors in humans or animals during or after a disaster as a disposition.

Injury, damage, or effect is the specialization of the disposition class. Parasite infection, fungus, bacteria, etc., are generic continuant. Generic entities are dependent on more than one entity for their existence. It allows migration from one bearer to another. Two large families of such entities exist: the Information artefact domain and biological sequence.

DMO upper class of role contains externally grounded realizable entities which come into action when an object feels change due to an externally bounded environment. This environment can relate to some people or any matter. A role specifies a purpose that is not essential to the object’s design but can be carried out. For example, a person can have many roles, such as a mother, a sister, a daughter, or some work role as an engineer or professor. State-level observation at any time and at any phase is the responsibility of the state commissioner of relief. The notable point is that the state commissioner of relief is a role in BFO, which is connected through state Level Observational Responsibility to other process entities.

A process is an occurrence that contains some activity that occurs or happens over time and has distinct ends. Entities in this category may have temporal parts, which may involve gaining or losing fragments. All disasters, including man-made and natural disasters, are suitable examples of the process. Hazard occurs for a specific amount of time and has a distinct beginning and end. Two distinct time stamps of the process have distinct spatial effects. DMO is packed up with processes, and disaster management without processes is like a pen without ink. Some processes are risk reduction, environmental impact assessment, monitoring, evaluation, water resource management, and so on.

**Figure 3:**
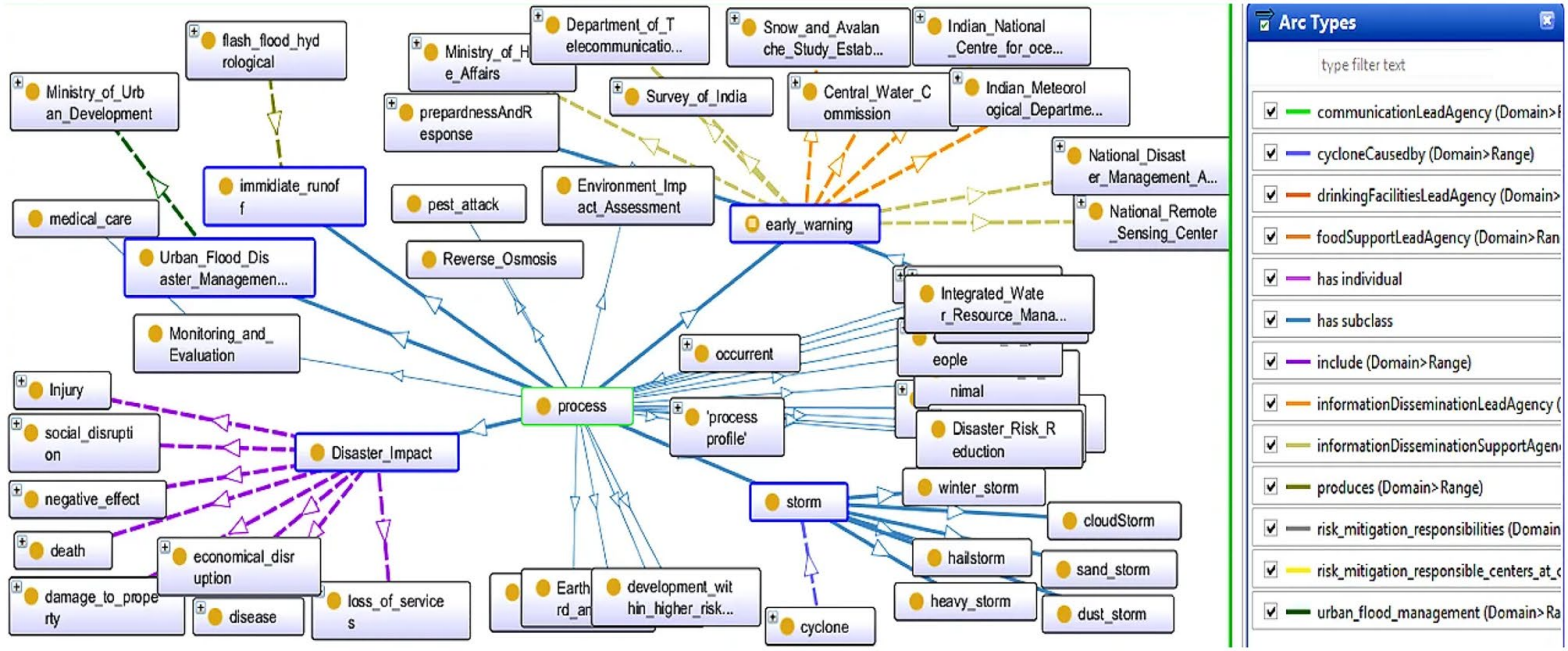
A snapshot from Protégé depicts entities as Process.

Every process has a beginning and an end. That starting and ending point is a zero-dimensional temporal region in terms of time, but it becomes the process boundary in terms of space. It is an occurrent entity that serves as the process’s instantaneous temporal boundary. All entities in this category are understood to refer to change that occurs spontaneously rather than as a result of such change. Process boundaries include death, birth, accidents, collapse, and so on. Figure 3 presents all properties and connectivity of process impact in DMO. Because the Process class contains a large number of sub-classes for this domain, it is impossible to expand all of them, so only a few are expanded.

Table 2 depicts the features of DMO in terms of class, properties, individuals, and expressivity captured by the axioms.

**Table 2:** DMO features.

Feature	Value
Class count	684
Object property count	159
Data property count	25
Individual count	24
Axiom count	3177
DL expressivity	ALCRF(D)

## Implementation and result analysis

Natural or man-made disaster causes are further classified into different categories based on the facts of eventuation and effect. Hydrological, meteorological, geophysical, climatological, and biological classifications are all possible. This article includes all disaster phase responsibility matrices and assistance support systems. Due to the domain’s complexity, it is extremely difficult to present all calamity case studies, so cyclone (a meteorological category) is taken as an instance to represent various cases.

### Causes and effects of cyclone

**Figure 4:**
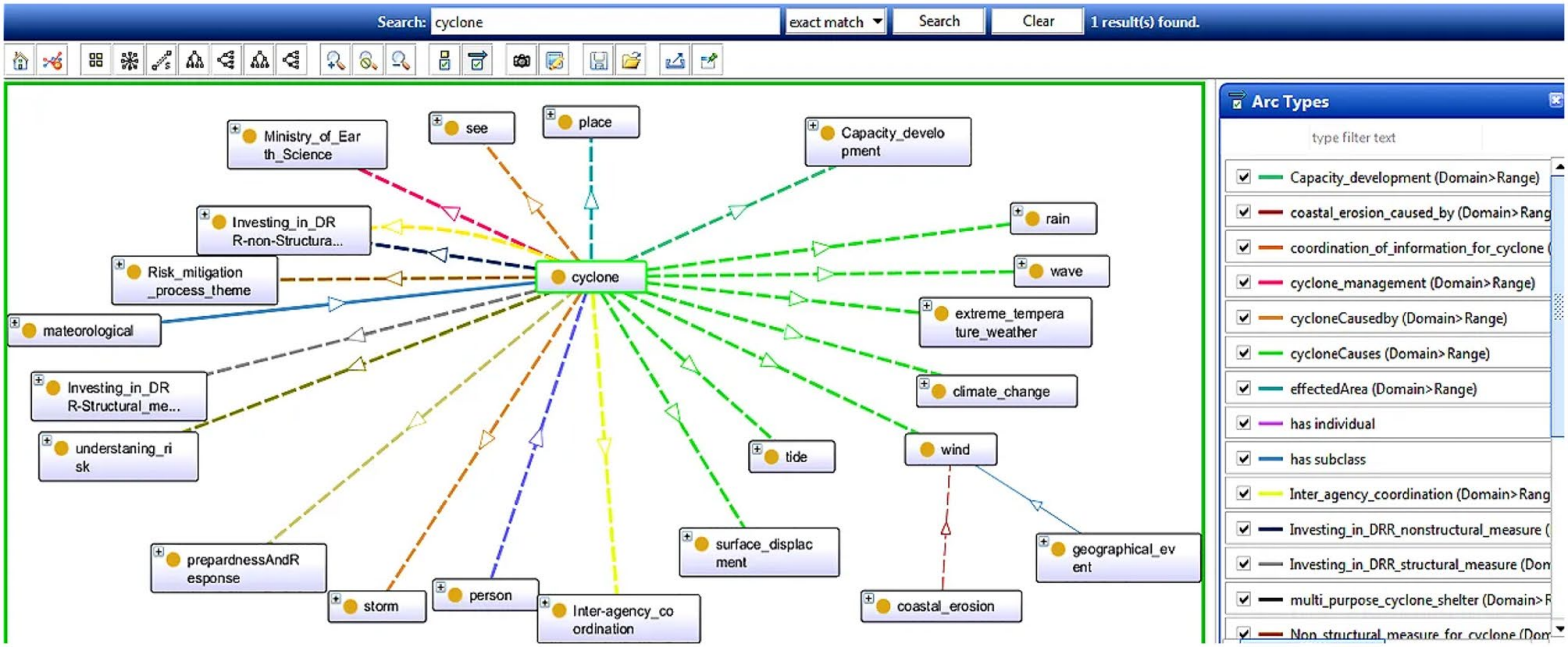
A snapshot from Protégé depicts causes and effects of cyclone.

According to the Indian calendar, cyclones occur in two seasons: October-November and May-June. During these months, the sea level rises dramatically, causing rapid climatic changes. Nearly India’s one-third population falls under the fate of cyclone-related hazards. Figure 4 presents the OntoGraph of cyclones under DMO. The different coloured arcs represent different properties relating to two entities. For example, the green arc represents cyclone causes whose domain cyclone range includes heavy rain, the wind of different speeds, sudden climate change, sea wave with defined speed and pressure, and surface displacement. The presented OntoGraph is very easy to understand. A tsunami may also lead to a cyclone, accompanied by heavy wind with a defined speed based on area and heavy or light rain. Ministry of Earth Science holds the responsibility for cyclone mapping and management.

### Risk mitigation for cyclone effects

The cyclone risk reduction and mitigation plan uphold the task of merging the significant themes in a nationwide attempt. Under the mitigation process, the risk of disaster is going to be reduced or eliminated. Any hazard can trigger other hazards. For example, a tsunami can produce landslides or floods. In the same way, cyclones generally lead to floods and various other events apart from primary hazards. The mitigation phase of DMO focuses on capability development for multiple hazards and their waterfall effects. The process of understanding risk prevents any hazard from transforming into a serious disaster. There are four primary criteria for mitigation:

- Inter-Agency Coordination
- Investing in DRR (Disaster Risk Reduction)-Structural Measures
- Investing in DRR (Disaster Risk Reduction)-Non-Structural Measures
- Capacity Development

### Understanding risk

Understanding disaster risk is the most important focus of all domains of action. The following are the primary components of activities:

- Observing all Networks of different centers
- Observing Information Systems, Forecasting, and Research
- Mapping/Zoning
- Systems Hazard Risk and Vulnerability
- Monitoring and Warning
- Dissemination of Information, Warnings and Data

These major themes have a wide range of responsibilities shared by the state and federal government^3^.

### Inter-agency co-ordination

Inter-agency interoperability is the heart of governing disaster risk. The necessary actions to improve the other significant responsibilities require synchronization and proper coordination among all nodal ministries of the respective theme. The agencies, departments of ministries, and government organizations are loaded with the hazard-specific duty of the government or those who are assumed to carry a major load in the matrix concerning the thematic area. This synchronization involves disseminating data among different branches, coordinating technical input, creating or improving rules, etc.

**Figure 5:**
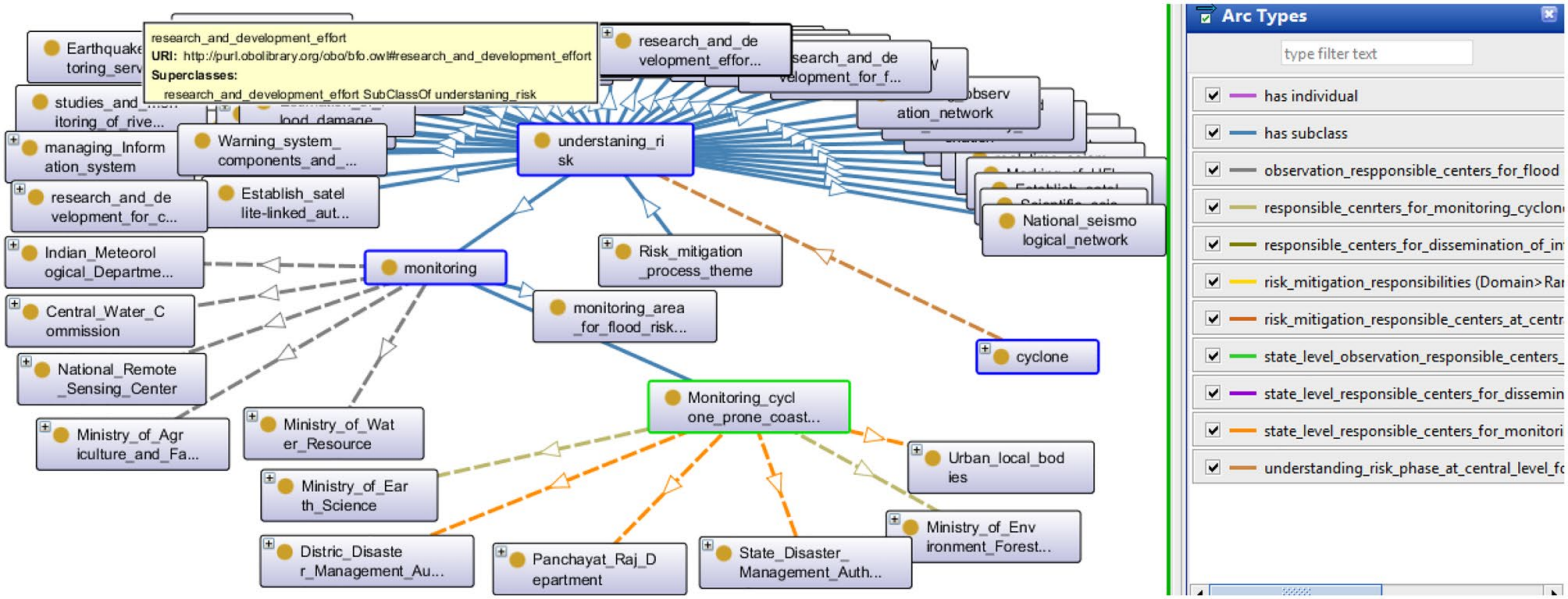
A snapshot from Protégé depicts monitoring under Inter-agency co-ordination.

Figure 5 depicts the Onto-graph for class monitoring. This Onto-graph can quickly locate responsible authorities for monitoring cyclone-prone coastal areas. In this case, the state disaster management authority, Panchayat raj department, district disaster management authority, and urban local bodies are responsible for the same.

### Investing in DRR-Structural measures

**Figure 6:**
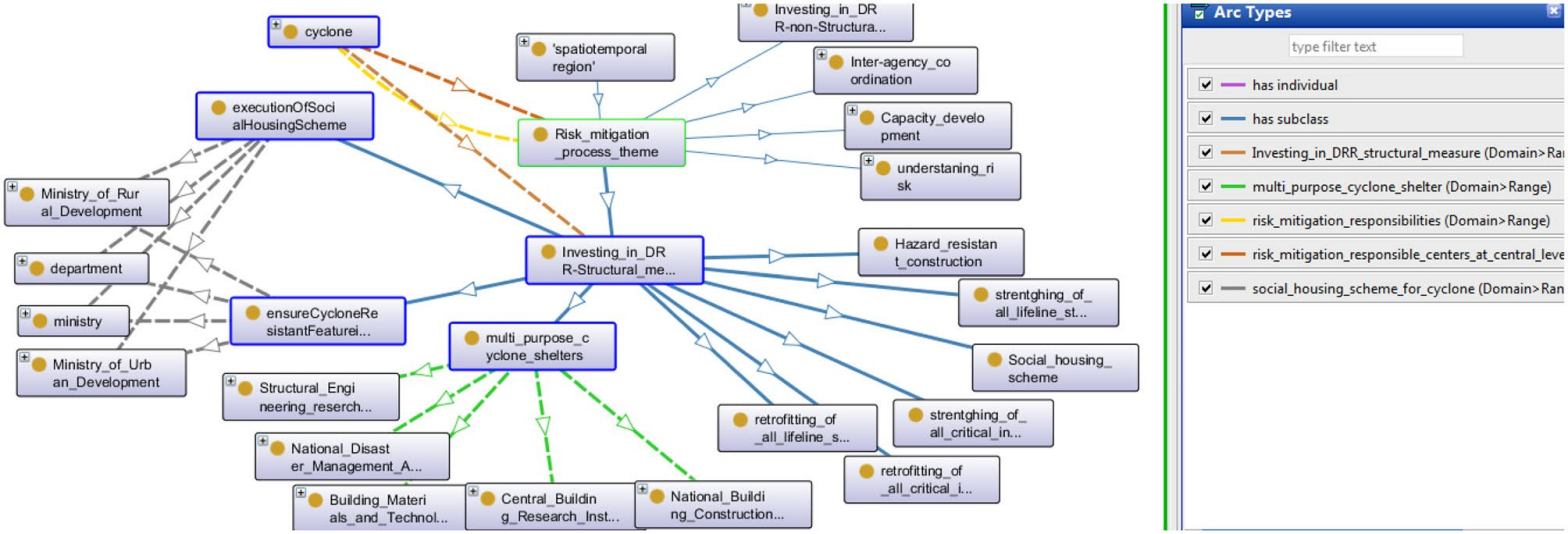
A snapshot from Protégé depicts DRR structural measures.

This area includes obligatory physical and structural steps. These subsist of diverged infrastructure and facilities compulsory to fulfil safety from the external environment in an emergency. Under this category, hazard resistance shelter construction, multipurpose house construction, and basic lifeline needs are implemented. The onto-graph presented in Fig. 6 contains further classifications as Multi-Purpose Cyclone Shelter, ensure Cyclone Resistant Features, retrofitting of all lifelines, execution Of Social Housing Scheme, etc. Here cyclone is connected with the risk mitigation theme, and Investing in DRR structural measure by brown arc denotes investing in DRR-structural measure relation.

### Investing in DRR-non-structural measures

**Figure 7:**
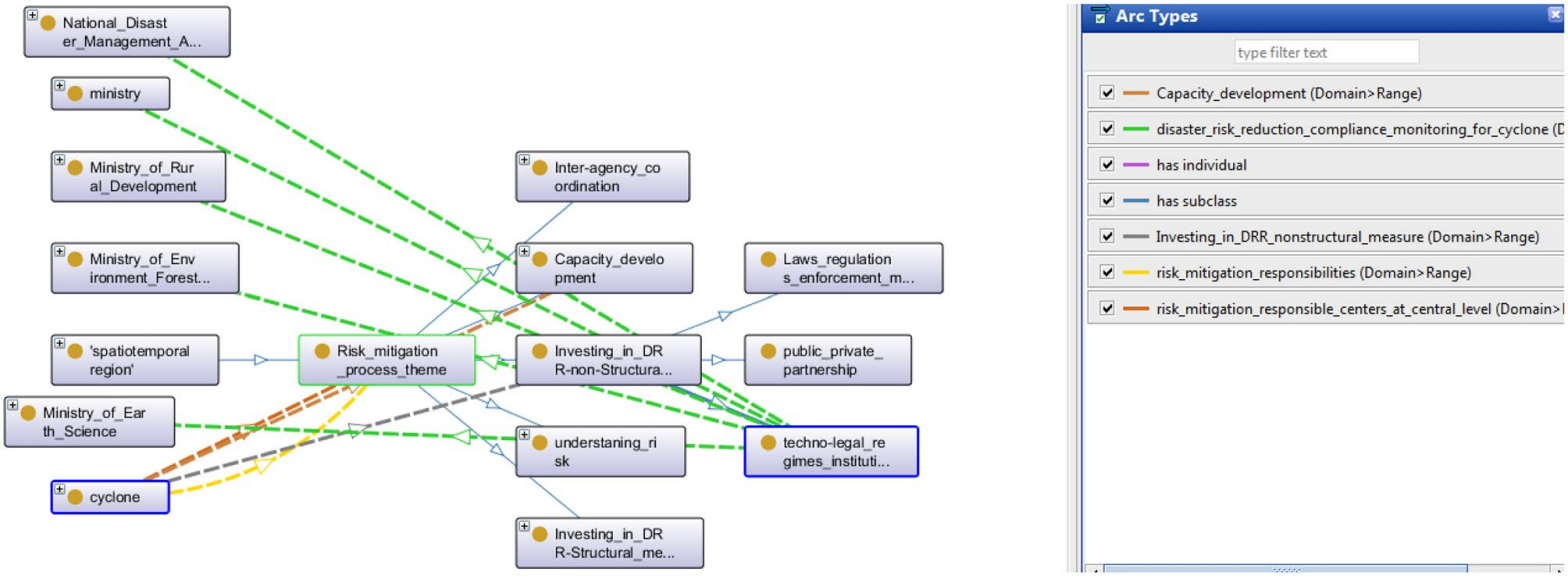
A snapshot from Protégé depicts DRR Non-Structural measure.

Non-structural measure includes all those factors whose presence can only be realized when provoked. It contains all the laws, rules, guidelines, and mechanisms to empower the risk control mechanism. It also initiates public and private cooperation guidelines. Figure 7 describes the onto-graph for Non-Structural measures in DMO.

### Capacity development

**Figure 8:**
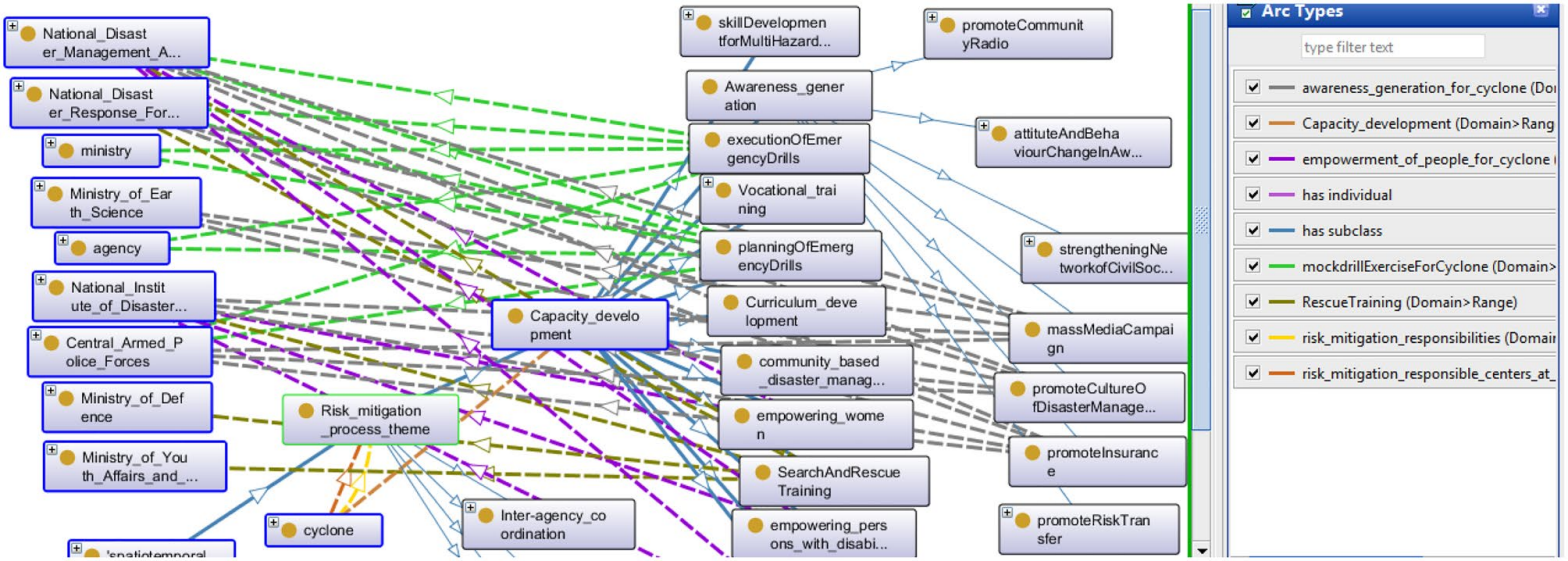
A snapshot from Protégé depicts the capacity development matrix.

Capacity development upgrades the resources available to reduce the risk of calamity. Under this, different training programs for awareness generation are conducted and train women, men, children, and persons with disability. The capability to map and implement various disaster mitigation phases must be empowered at all stages, from the basic to the higher standard of governance. Figure 8 presents the capacity development matrix of DMO.

### Response and recovery for cyclone

**Figure 9:**
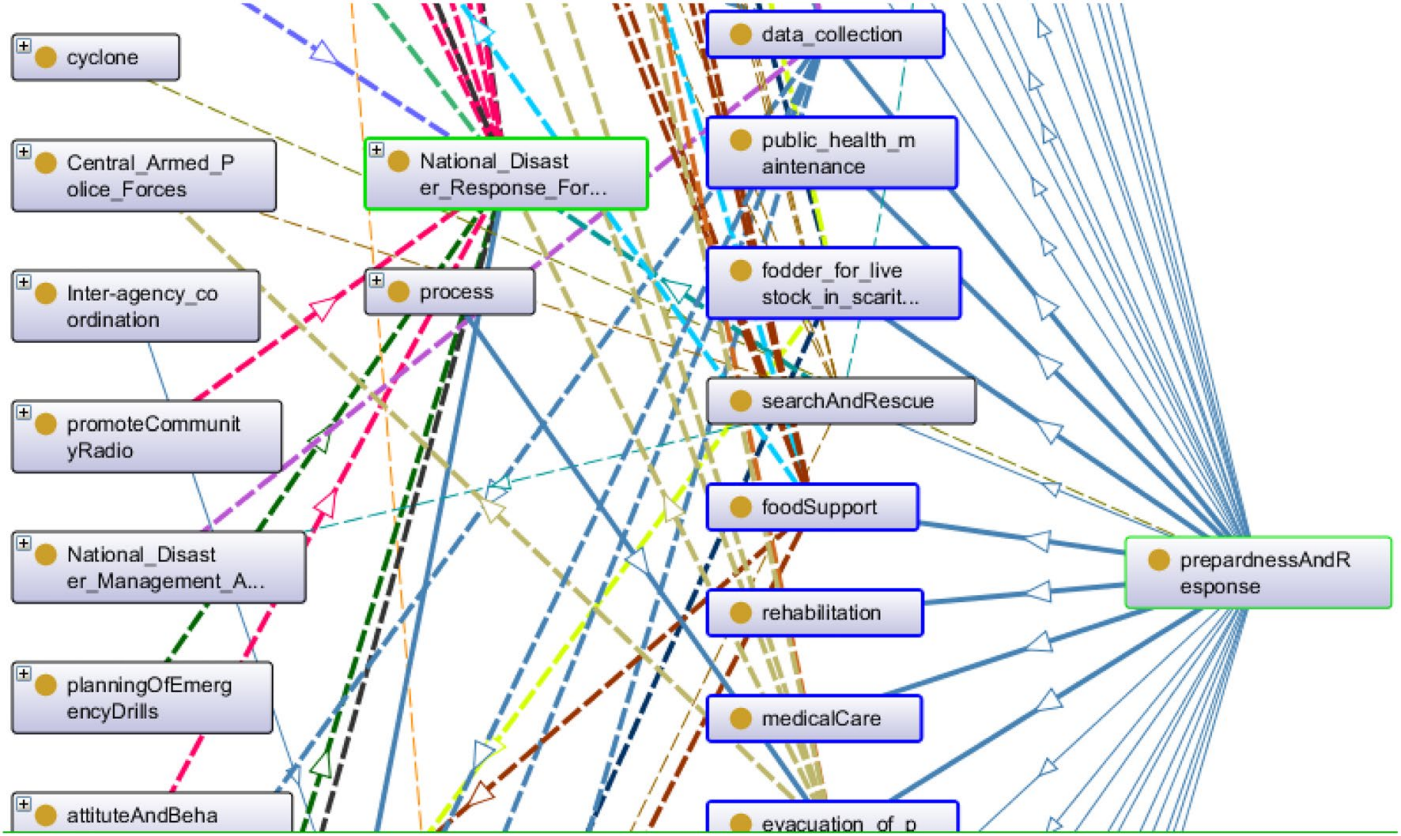
A snapshot from Protégé depicts the response and recovery matrix.

When a disaster strikes a region, it affects the entire way of life. The first government step is to provide safety to those who may become entangled in the thread or require immediate assistance. The secondary theme is to provide basic survival requirements. These survival needs include food and lodging, communication channel, transportation, sanitation, medical care, etc. Search and rescue operation of people and animals from the stroked area is one of the major responsibilities of authority. DMO provides a platform where all authorities can investigate “who will do what”. This way, confusion about any activity and respective responsible authority gets eliminated. Figure 9 describes the onto-graph for the response and recovery matrix.

### Querying the DMO

The government has categorized different norms of assistance, including gratuitous relief assistance, search and rescue assistance, relief measures, assistance, clearance of affected area assistance, agriculture assistance, animal husbandry resistance, fishery assistance, handicrafts/handloom assistance, housing, and infrastructure assistance. This section elaborates on implementing DMO guidelines to aid disaster-affected people of different categories using SWRL^41^ (Semantic Web Rule Language). SWRL is based on OWL-DL^42^ (Description Logic) and Horn Logic. This article performed a subjective assessment of the proposed disaster management ontology. It used the National Disaster Management Plan (NDMP) as a reference for assessing the recovery assistance provided by the proposed ontology. This study randomly selected several disaster scenarios and assessed the ontology’s ability to provide appropriate recovery assistance in each case. One scenario, for example, involves a cyclone that damages homes and infrastructure in a coastal town. It assessed the ontology’s ability to provide appropriate recovery assistance to those affected. In another scenario, this study assesses ontology’s ability to provide appropriate medical assistance to cyclone victims. The article assessed the effectiveness and usefulness of the proposed ontology in providing recovery assistance in various disaster scenarios by conducting this subjective assessment. This assessment enabled us to identify areas for improvement and make the necessary changes to ensure the ontology’s effectiveness in real-world disaster management situations. In this article, DMO Reasoner covers the two major themes listed below:

- Determines an individual’s details, such as his location and family.
- Calculates assistance for disaster victims based on the loss of their body parts, property, business, etc.

**Figure 10:**
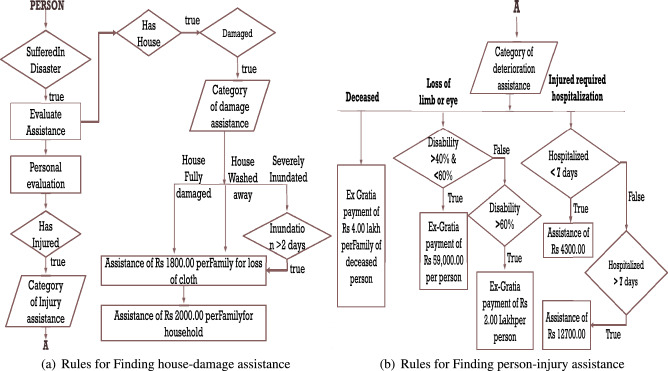
Flowchart of the rules for getting assistance for disaster victims.

In this section, we considered two scenarios for household and gratuitous assistance, which are depicted in the flow chart. This allows the rule expert to develop rules in the correct flow, revealing various aspects of any given category or division. Figure 10a depicts a flowchart if a person owns a home and is impacted by the disaster. Figure 10b depicts the flowchart, which is linked to Fig. 10a by label A. It eliminates inconsistencies in output across various dilemmatic situations. For instance, if a person is hospitalised for more than seven days, has lost his eyes due to an injury, and has lost his life during treatment. Three cases are mentioned here, each with its own SWRL rules. Since the person falls into all three scenarios mentioned, what should the assistant amount be? DMO eliminates such ambiguity by enforcing one or more rules. This article presents a sample set of seventeen rulesets for the three scenarios: family assistance, household assistance, and disaster victim assistance. For example, rulesets 1, 2, 3, 4, 5, 6, 7, 8, and 9 provide sample rules for Figure 10(b) that depicts sample victim assistance, and rulesets 10, 11, 12 and 13 are sample rules for Figure 10(a) that provides sample house-damage assistance.

#### Finding details of a person

This article provides rulesets for inferring family relationships and individual addresses. This can aid in the integration of various systems and resources, allowing for more effective decision-making and resource allocation during disaster relief efforts. Ontology rules can automate the process of identifying family members and eliminate the need for manual searching and analysis, which is especially important when direct family member identification is impossible or difficult due to various factors such as missing or incomplete data. These rules help decision-makers make more informed and effective decisions about resource allocation, emergency response, and recovery efforts by providing accurate and comprehensive information about family members. They also improve the mental health outcomes of affected people by facilitating reunification. Also, during the recovery phase, it is necessary to provide resources such as financial assistance, shelter, and medical care to those affected by the disaster. In disaster situations, where resources are often limited, assisting with recovery is critical, and assistance allocation decisions can significantly impact individuals and communities. This article proposes a set of rules for automating the family identification procedure. If the rescue team finds an injured or deceased person while on the scene, the DMO can provide the necessary information. For example, if Amitabh is found injured, DMO will notify the system that Jaya is his wife and his home address and other pertinent information. Similarly, if a child is found, information about his parents and residential address will be provided. These outcomes are based on previously implemented ground facts in the knowledge base, and the rule engine operates on these facts. The sample rules used here are given in ruleset 14, 15, 16, and 17.

1 $$\begin{aligned}{} & {} person(?p) ^\wedge sufferedInDisaster(?p, ?d) ^\wedge disaster(?d) ^\wedge lossOfLimb(?p, true) ^\wedge disability(?p, ?x) ^\wedge \nonumber \\{} & {} \quad swrlb:greaterThan(?x, 60) -> exGratiaPayment(?p, 200000) \end{aligned}$$

2 $$\begin{aligned}{} & {} person(?p) ^\wedge sufferedInDisaster(?p, ?d) ^\wedge disaster(?d) ^\wedge grievousInjured(?p, true) ^\wedge requiredHospitalization(?p, ?y) ^\wedge \nonumber \\{} & {} \quad swrlb:lessThan(?y, 7) -> assistance(?p, 4300) \end{aligned}$$

3 $$\begin{aligned}{} & {} person(?p) ^\wedge sufferedInDisaster(?p, ?d) ^\wedge disaster(?d) ^\wedge grievousInjured(?p, true) ^\wedge requiredHospitalization(?p, ?y) ^\wedge \nonumber \\{} & {} \quad swrlb:greaterThanOrEqual(?y, 7) -> assistance(?p, 12700) \end{aligned}$$

4 $$\begin{aligned}{} & {} person(?p) ^\wedge sufferedInDisaster(?p, ?d) ^\wedge disaster(?d) ^\wedge lossOfEye(?p, true) ^\wedge disability(?p, ?x) ^\wedge \nonumber \\{} & {} \quad swrlb:greaterThan(?x, 60) -> exGratiaPayment(?p, 200000) \end{aligned}$$

5 $$\begin{aligned}{} & {} person(?p) ^\wedge sufferedInDisaster(?p, ?d) ^\wedge disaster(?d) ^\wedge lossOfEye(?p, true) ^\wedge grievousInjured(?p, true) ^\wedge \nonumber \\{} & {} \quad deceased(?p, true) -> exGratiaPayment(?p, 400000) \end{aligned}$$

6 $$\begin{aligned}{} & {} person(?p) ^\wedge sufferedInDisaster(?p, ?d) ^\wedge disaster(?d) ^\wedge lossOfEye(?p, true) ^\wedge disability(?p, ?x) ^\wedge \nonumber \\{} & {} \quad swrlb:lessThanOrEqual(?x, 60) ^\wedge swrlb:greaterThanOrEqual(?x, 40) -> exGratiaPayment(?p, 59100) \end{aligned}$$

7 $$\begin{aligned}{} & {} person(?p) ^\wedge disaster(?d) ^\wedge sufferedInDisaster(?p, ?d) ^\wedge lossOfLimb(?p, true) ^\wedge disability(?p, ?x) ^\wedge \nonumber \\{} & {} \quad swrlb:lessThanOrEqual(?x, 60) ^\wedge swrlb:greaterThanOrEqual(?x, 40) -> exGratiaPayment(?p, 59100) \end{aligned}$$

8 $$\begin{aligned}{} & {} person(?p) ^\wedge sufferedInDisaster(?p, ?d) ^\wedge disaster(?d) ^\wedge lossOfLimb(?p, true) ^\wedge grievousInjured(?p, true) ^\wedge \nonumber \\{} & {} \quad deceased(?p, true) -> exGratiaPayment(?p, 400000) \end{aligned}$$

9 $$\begin{aligned}{} & {} person(?p) ^\wedge sufferedInDisaster(?p, ?d) ^\wedge deceased(?p, true) ^\wedge disaster(?d) -> exGratiaPayment(?p, 400000) \end{aligned}$$

10 $$\begin{aligned}{} & {} person(?p) ^\wedge house(?h) ^\wedge hasHouse(?p, ?h) ^\wedge sufferedInNaturalCalamity(?h, true) ^\wedge houseWashedAway(?h, true) \nonumber \\{} & {} \quad -> houseHoldGoodsAssistancePerFamily(?p, 1800) ^\wedge clothsAssistancePerFamily(?p, 2000) \end{aligned}$$

11 $$\begin{aligned}{} & {} person(?p) ^\wedge house(?h) ^\wedge sufferedInNaturalCalamity(?h, true) ^\wedge hasHouse(?p, ?h) ^\wedge houseSeverelyEnundated(?h, true) ^\wedge \nonumber \\{} & {} \quad houseEnoundationDuration(?h, ?y) ^\wedge swrlb:greaterThan(?y, 2) -> houseHoldGoodsAssistancePerFamily(?p, 1800) ^\wedge \nonumber \\{} & {} \quad clothsAssistancePerFamily(?p, 2000) \end{aligned}$$

12 $$\begin{aligned}{} & {} person(?p) ^\wedge house(?h) ^\wedge hasHouse(?p, ?h) ^\wedge sufferedInNaturalCalamity(?p, true) ^\wedge houseFullyDamaged(?h, true) -> \nonumber \\{} & {} \quad houseHoldGoodsAssistancePerFamily(?p, 1800) ^\wedge clothsAssistancePerFamily(?p, 2000) \end{aligned}$$

13 $$\begin{aligned}{} & {} areaHasHouse(?p, ?h) ^\wedge disaster(?d) ^\wedge place(?p) ^\wedge house(?h) ^\wedge effectedArea(?d, ?p) -> \nonumber \\{} & {} \quad sufferedInNaturalCalamity(?h, true) \end{aligned}$$

14 $$\begin{aligned}{} & {} person(?p1) ^\wedge person(?p2) ^\wedge person(?f1) ^\wedge hasMother(?p1, ?f1) ^\wedge hasMother(?p2, ?f1) ^\wedge person(?h)\nonumber \\{} & {} \quad ^\wedge hasHusband(?f1, ?h) -> hasSibling(?p1, ?p2) ^\wedge hasFather(?p1, ?h) \end{aligned}$$

15 $$\begin{aligned}{} & {} person(?p1) ^\wedge father(?f1) ^\wedge isaFather(?p1, ?f1) ^\wedge child(?c1) ^\wedge person(?p2) ^\wedge isaChild(?p2, ?c1) ^\wedge haschild(?p1, ?p2) ^\wedge \nonumber \\{} & {} \quad person(?p3) ^\wedge mother(?m1) ^\wedge isaMother(?p3, ?m1) ^\wedge haschild(?p3, ?p2) -> hasWife(?p1, ?p3) ^\wedge hasHusband(?p3, ?p1) \end{aligned}$$

16 $$\begin{aligned}{} & {} person(?p) ^\wedge person(?w) ^\wedge house(?h) ^\wedge hasWife(?p, ?w) ^\wedge hasHouse(?p, ?h) -> hasHouse(?w, ?h) \end{aligned}$$

17 $$\begin{aligned}{} & {} person(?p) ^\wedge haschild(?p, ?c) ^\wedge hasWife(?p, ?w) ^\wedge person(?w) ^\wedge person(?c) -> haschild(?w, ?c) ^\wedge hasMother(?c, ?w) \end{aligned}$$

**Figure 11:**
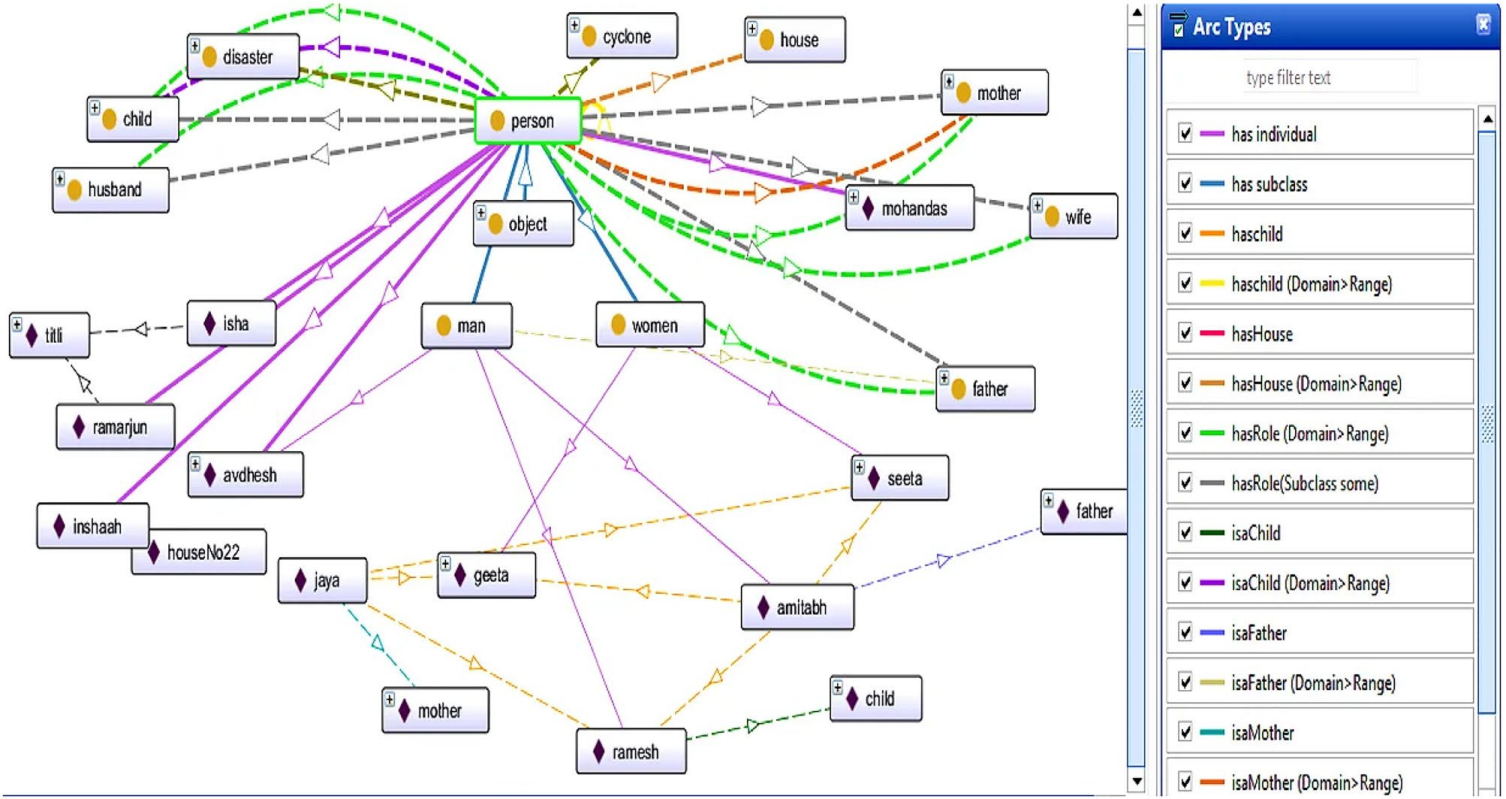
A snapshot from Protégé depicts family relation Onto-graph.

Figure 11 presents family relations via instances, and different coloured arcs present the type of relations. The yellow arc represents the Is a Child property, which connects Ramesh, Geeta, and Seeta with a child, as an instance of role class. Amitabh is a class person and plays the roles of father and husband. Jaya is a woman who plays the roles of mother and wife. Amitabh has three children, Ramesh, Geeta, and Seeta, and Jaya is his wife. In this case, a reasoner is required to calculate Jaya’s relationship.

**Figure 12:**
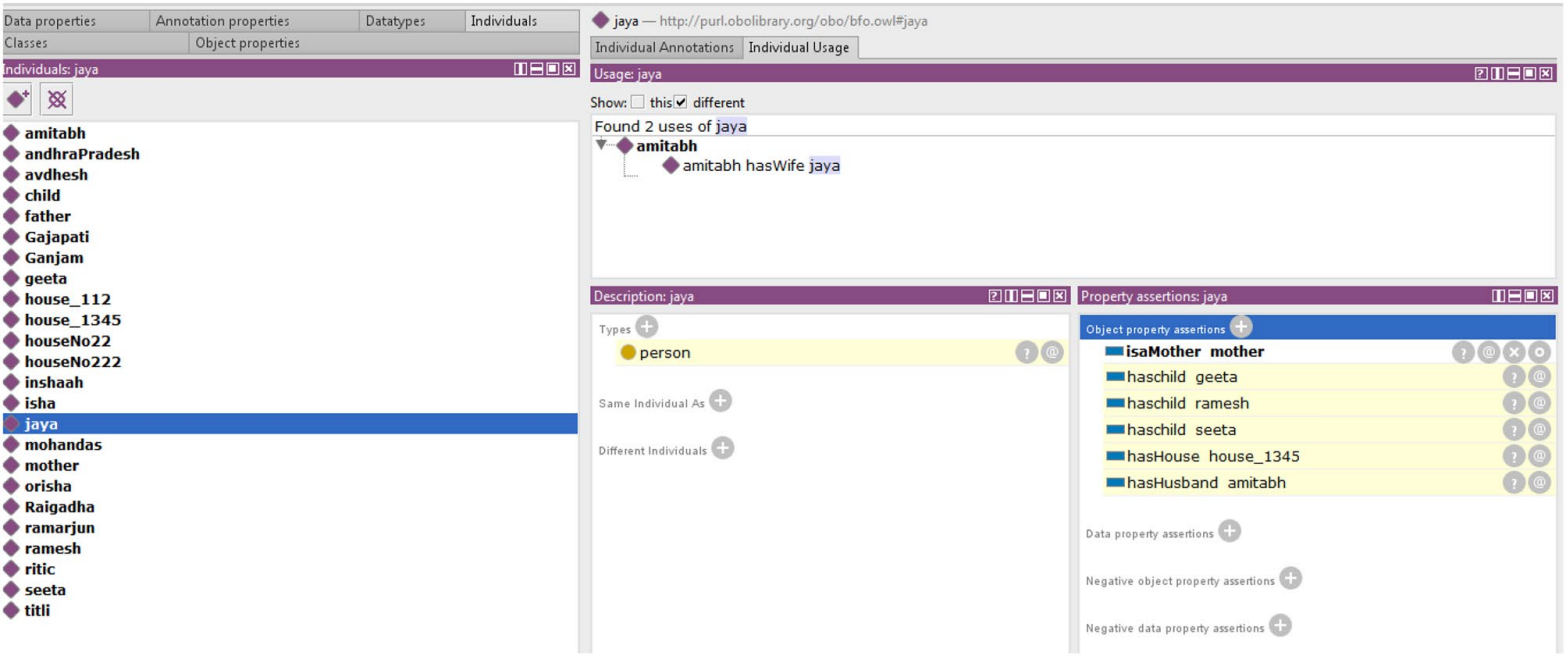
A snapshot from Protégé depicts the reasoner tracing family detail of a person jaya.

In Fig. 12, the reasoner evaluates the family details of Jaya. The reasoner says that Geeta, Ramesh, and Seeta are Jaya’s children, Amitabh is Jaya’s husband, and Jaya has house no 1345.

#### Assistance calculation

The Indian government^3^ has established some guidelines and rules to assist disaster victims who meet certain criteria. A person who is primarily injured or deceased is eligible for free assistance. The severity and intensity of the injury determine the assistance amount, whereas property damage is covered by domestic assistance. The magnitude of destruction and loss of property are prime factors in determining household assistance per family. As a disaster damages or destroys public property including government schools, hospitals, public transport, roads and bridges. Reconstruction or repair of these public properties comes under the infrastructure assistance category. This article proposes a set of rules to aid in the automation of the recovery procedure, which can aid in improving the accuracy and transparency of disaster assistance calculations. In disaster situations, where resources are often limited, assisting with recovery is critical, and assistance allocation decisions can have a significant impact on individuals and communities.

**Figure 13:**
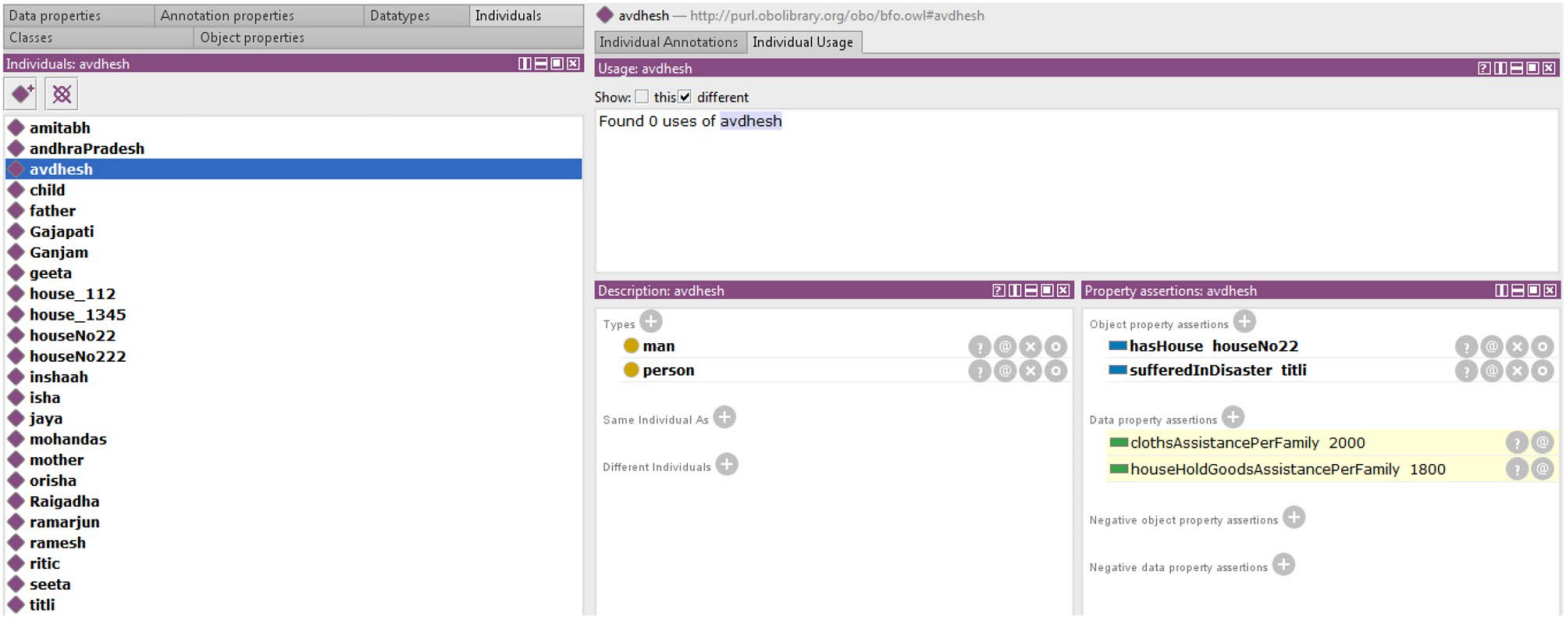
Reasoner finding household assistance.

In Fig. 13, Avdhesh is a particular class of person. As input Avdhesh has house houseNo22, located in a cyclone-affected area. The Reasoner will be fed about cyclone-affected areas. If houseNo22 is located in any of those areas, that house is also disaster-affected; the kind of effect is then externally evaluated. Here houseNo22 is destroyed, so cloth assistance and household goods assistance are calculated.

**Figure 14:**
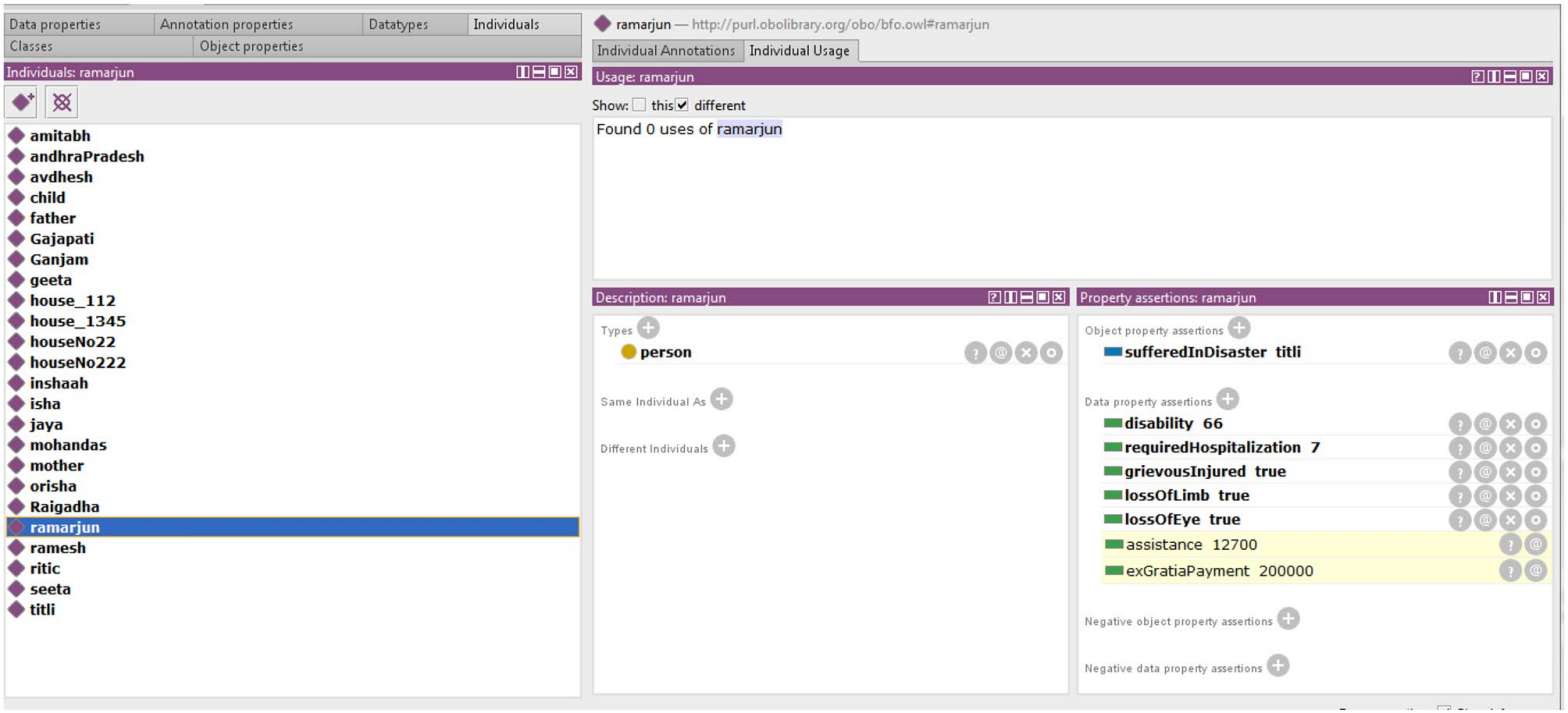
Reasoner finding household assistance.

**Figure 15:**
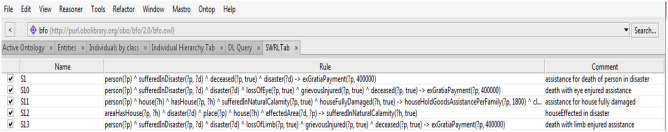
Rules for injured individuals.

Figure 14 describes the assistance calculation for Ramarjun, who has been injured and hospitalized for more than seven days. He had lost an eye and is 66% disabled. Ramarjun will find the assistance of 12,700 and 200,000 for their respective reasons. Figure 15 depicts the rules that were used.

### Discussion

It wasn’t easy to decide the category of entities during the concept classification. BFO assigns classification and believes that anything that exists can be classified. For example, emotions, rules, laws, and policy are concepts whose category is highly obligated to a specific concept of BFO. At first glance, it isn’t easy to decide which class of BFO these concepts belong to. The desired class of concepts is decided by investigating the characteristics, property, and relation to other entities. While designing the knowledge base, the next challenge was distinguishing those object properties whose domain and range were differentiated from the same object property name. In ontology, every concept, including entities and properties, has IRI (International Resource Identifier), which provides a unique identity to each concept, and repetition is restricted to core concepts, so if with the same property name, different boundaries exist. The property name must include the purpose or subject of the property. For illustration, ‘causedBy’is an object property name in DMO. Still, in this domain, causedBy can infer many purposes like landslidecausedBy, cyclonecausedBy, etc., with respective domains and ranges which are not a proper part of another one. In the case of the given an example, the unique object properties are landslideCausedBy and cycloneCausedBy. Apart from those listed, two challenges, including the development of the rule in OWL-DL, were faced during the design of DMO and were tackled with a deep study of respective concepts. Using similar data modelling, a structured knowledge flow for different other hazards can be prepared. DMO can be used to deduce implicit knowledge and to arrive at a particular decision. An Inference engine may be proposed to extend the ontology, which is helpful in collaborating with other ontologies for various purposes.

In the future, there is a lot of room to grow the Disaster Management Ontology. We can integrate the ontology with other systems, such as GIS or emergency response management systems, to enable more effective decision-making. Integrating ontology with natural language processing tools can enable more flexible querying and natural interactions. Testing and validating the created rules and ontology in real-world disaster scenarios can evaluate their effectiveness and identify areas for improvement. Finally, the ontology can be further enhanced to provide more personalized and targeted recovery assistance to affected people based on their specific needs and circumstances.

## Conclusion

Ontologies are now used in information science as a client tool for performing tasks like enhancing communication between user and machine. As a result, the semantic web’s basic concept has matured to a higher level, and different ontologies have been designed. The BFO supports reasoning along with the general open-world semantics in a domain-neutral manner among different categories and classifications. The proposed Decision Support System combines Knowledge Base with SWRL to get a more desirable and logic-based solution, considering the domain of Disaster Management. The proposed Ontology-based Decision Support System is made to provide betterment in different phases. This helps in the smooth flow of knowledge across the different levels of authority. This knowledge base includes the organizational structure of the Indian culture to handle any disaster. Developed Ontology can represent an entire knowledge base in user-friendly ontology graphs. The responsibility matrix of different disaster management phases and assistance of funds to disaster effect people suggested by the NDMP through its various guidelines has been implemented using SWRL. The inclusion of SWRL rules for implementing the assistance is the actual heart and soul of our decision support system. The decision-making task, which lies in the hand of the DMO national disaster response fund team, is most likely to get valuable assistance from our proposed system. These rules allow for the outcomes of any affected person’s family investigation and various other NDMP-induced activities. We randomly selected several disaster scenarios and evaluated the ontology’s ability to provide appropriate recovery assistance in each case. This evaluation allowed us to identify areas for improvement and make the necessary changes to ensure the ontology’s effectiveness in real-world disaster management situations. Various actors involved at different levels are the most likely decision-making authorities to draw benefits in decision-making.

## Data Availability

The datasets used and/or analysed during the current study are available from the corresponding author on reasonable request.
